# Notes of Four Cases of Leprosy in Minnesota

**Published:** 1879-02

**Authors:** Chr. Gronvold

**Affiliations:** Norway, Minn.


### NOTES OF FOUR CASES OF LEPROSY IN MINNESOTA.

By CHR. GRONVOLD, M.D., Norway, Minn.

### I. ANÆSTHETIC FORM OF ELEPHANTIASIS GRÆCORUM.

Christen Johnson Hauglum, native of Arnfjord, Sogn, Norway, came to America in 1861, and settled in Long Prairie, Illinois. Eight weeks afterwards, he went to Goodhue County, Minnesota, where he is now living, in the town of Wanamingo.

When he was 24 years old, he was hale, hearty, light-footed and sharp-sighted, and had never been ill. He had been accustomed both to fishing in the sea and hunting in the mountains. He only remembers that during the spring of each year he would be troubled for some days with an eruption upon the lower part of the face, which would itch, and often result in soreness of the surface from scratching. This was, however, of small moment, though it has recurred with tolerable regularity to the present date.

In 1856 he was made ill, as he thinks, by exposure to cold during his excursions upon the sea and on the mountains. He was then confined to his bed for seven weeks, and for some time afterwards was unable to do any work. The disease began with chills, and was characterized by heated skin, headache, pain, generalized and finally located in the left thigh, with a disposition to stretch the limbs, heaviness, drowsiness and insomnia for several days and nights. This was succeeded by a sleep which lasted without interruption night and day for several days. Then he had a cough, from which he still suffers, with distressing dyspnœa and abundant hæmoptysis, the latter symptom often returning since the date mentioned. After this attack he seemed to be quite sensitive to cold, but declares that in other respects he was perfectly well.

He found, however, that once or twice yearly he would suffer from similar attacks, each accompanied by severe pain in the left thigh, usually in the spring of the year, when the ice was melting, but frequently also in the fall, when the frost set in. He would, on these occasions, generally be confined to his bed for from three to eight days, feeling for some time afterwards very uncomfortable with anorexia and debility. Neither at this time nor for years afterward did he suffer from symptoms of cutaneous disease, such as tingling, pricking, anæsthesia, or pemphigoid lesions.

During his voyage across the Atlantic, in 1861, he had an attack of shivering and fever, more severe than any heretofore experienced. He recovered so far as to be able to work in the harvest-field when he reached Illinois, but later had such severe cough, dyspnœa and hæmoptysis that he thought he would soon die. He however recovered sufficiently to remove to Minnesota.

Here his attacks, though recurring once or twice annually, were less prolonged and severe than formerly. The shivering fits would last for several hours, but when the fever and pain were relieved he would regain his appetite and feeling of general well-being with promptness, and without the periods of malaise heretofore experienced. In a few years, however, his attention was attracted to an increasing numbness in several parts of the extremities. In 1870, sensation had considerably diminished on the peroneal side of the legs and on the ulnar side of the forearms, and corresponding surfaces of the hands.

In 1871, excoriations appeared upon the nates, degenerating into sores, which did not heal for three months.

In 1872, the hair of the eyebrows disappeared, followed in the ensuing year by the eyelashes.

In June of 1876 the feet and legs commenced to swell, and the tumefaction, in the course of two months, involved, to a great degree, the entire lower extremities, the scrotum, and, to some extent, the arms and hands. All the swollen parts were more or less anæsthetic, but this symptom was more marked on the left side. He was in the habit of pricking the skin with a needle to relieve the tension, and from the punctures thus produced there immediately flowed a copious, limpid, yellowish fluid, in quantity sufficient to form small streams. Under the influence of warm cataplasms, this discharge would continue during the night, and in the morning considerably diminish the size of the extremities. Often the amount discharged in twenty-four hours would equal two quarts. He commenced to grow hoarse, and the old cough and dyspnœa returned with severity. By October the swelling had decreased to a considerable extent, but there was soreness of the affected parts, cephalalgia, failure of the eyesight, and frequent fits of shivering. In December the swelling had disappeared from all places except the left foot and a portion of the fingers and hands. The parts formerly swollen then became hyperæsthetic, so that the slightest touch caused pain.

During the winter he steadily improved, and by the ensuing summer he was able to move around again. Only the left foot was swollen, but a more complete and increasing anæsthesia succeeded to the former condition of hyperæsthesia.

In the fall of 1877 he had a severe recurrence of his former symptoms, of a character, however, somewhat different from that noted before. A severe headache, especially located above the root of the nose, was followed with a double acute iritis, severe pain, and a copious discharge. The eyes felt as if they were squeezed backward in their orbits, and this pain, when no atropia was used, would be continuous day and night; it did not completely disappear for the next nine months. Some time after the onset of this attack, a painful tumor occurred below and behind the left ear, which in four days opened, and is still discharging.

About fourteen days after this, in November of 1877, there appeared on the sides of the left foot six small bluish spots, soon followed by sloughing and exposure of the underlying bone. At the same time a bulla, as large as a twenty-cent-piece, appeared beneath the heel. This burst and exposed a dark red floor, which soon became black and fissured, the fissure extending to the os calcis. Further sloughing extended and deepened this, till an excavated ulcer was formed with the os calcis revealed at the bottom, discharging a stinking, ichorous, viscid matter. This communicated subsequently with other sores, which in a short time after healed. The ligaments were softened, almost disintegrated, and their connections so loose that the part of the foot in front of this sore seemed attached merely by the skin, and the os calcis so nearly disengaged that the nurse thought it would fall out, and tried to remove it, but found some posterior attachments remaining which held it in place.

At the present time (September, 1878), this bone has, on account of the weight of the foot resting upon it, been dislocated forwards, under and at the side of the other tarsal and metatarsal bones, so that the foot in front of the sore presents a very swollen and unshapely appearance. It is a mere insensitive bag of half-disintegrated bones and jelly-like muscles, between which the finger can be inserted to its whole length in different directions. The ulcer is oblong, three by two inches in extent, with callous undermined borders and a pallid slimy floor of muscles. An exposed articular facet of the astragalus has taken the place of the dislocated os calcis, which in its turn is moving forward, so that the ends of the tibia and fibula will soon appear at the bottom of the sore. The discharge is a thin limpid fluid, in quantity sufficient to drench the dressings, sheets, and mattress. As the foot is quite loosely attached, it can readily be carried up along the leg, a position it will assume whenever the foot is stretched and the toes pressed against anything.

At present he is almost without pain, owing possibly to the free discharge or to the periodicity of the disease. Walking is of course impossible, and he has not the strength to use crutches. Before the os calcis was dislocated he essayed to take a step, but the effort induced severe hemorrhage. The anæsthesia is complete to above the ankle, a few patches on the inside of the foot excepted. In the remaining portions of the same leg there is partial anæsthesia, especially below the knee and on the peroneal side; also in corresponding parts of the other leg and over the hand and forearms, chiefly on the ulnar side. The sensation in the lower side of the face is also somewhat blunted.

The skin of the affected parts is dry and withered, peeling off in thin layers; on the left leg it is thicker, resembling dried fish-skin. About the sore in the foot it is considerably thickened. Sweat is secreted only on the forehead and trunk, especially the upper part of the latter.

The general appearance is that of wasting and emaciation, with muscular atrophy, especially about the anæsthetic surfaces. Sight is much impaired in both eyes, especially the left, where the pupil is obstructed by deposits upon the capsule of the lens, and the pupillary margin exhibits the fringes resulting from iritis. The eyes also are moister than normal, and the lids are glued together every morning. The lids and brows are destitute of hair. The upper lids are thin; and he complains of a tendency to ptosis, especially upon the left side, when the eyelids have been raised for any length of time. The lower lids have lost their fullness in part, but the tarsal border is closely applied to the globe, and the lids cover the eyes well. The left inner canthus is especially large; the lachrymal caruncles have almost gone. The left half of the mouth is partially paralyzed, the lips being relaxed, though they shut together fairly well. The mucous membranes of the mouth and pharynx are pale, but in other respects normal, though there is occasional dysphagia. The voice is clear and natural, when not affected with "a cold." Hearing is impaired on the left side. He states that his sense of taste is normal, though it is probably impaired. He likes some articles; dislikes sweet and salted food, and complains frequently of a bad taste in the mouth. The appetite is good; the bowels regular. The Schneiderian membrane often feels to him dry, though he thinks his sense of smell is unimpaired. The fingers of both hands are bent into the palms; they are very thin, crooked, claw-like, and clubbed at the extremity; this latter appearance being due to the large, high-arched, rounded, flesh-colored, and inverted nails, which are almost continuous with the flesh, and are only recognizable as of different structure on close inspection. The nails of the toes, which have begun to fall off, are of similar appearance.

He has six or seven healthy children from 6 to 21 years old. Only one of his relatives, so far as he knows, is affected with a similar disease,—the brother of his mother's father.

If the patient's statements are correct regarding the development of his disease previous to 1873, when it was first brought under surveillance, it differs from those previously described by Professors Boeck and Daniellssen in this, that no pemphigoid eruption appeared until the later stages of the disease were attained.

*Case II.*—Peter Benonison Bistranden, born in 1843, in Losoten, Norway. His father's brother was leprous. Up to the year 1868 he was quite well. In 1869 he came to America on account of his health. Right leg anæsthetic. In 1876 he had acute iritis. In 1878 he died of anæsthetic leprosy.

III. TUBERCULAR FORM OF ELEPHANTIASIS GRÆCORUM.

Hjlmer Henrikson Dybaa, nativity in Vingvaagen, Vernes, Tnondhjem Stift, Norway, 1849, came to Zumbrota, Goodhue Co., Minn., in 1869. He did not suffer from disease, to his knowledge, before 1873, when, after some illness, he discovered tubercles and sores in several places upon the extremities. Every year he has suffered from attacks of indisposition. In January, 1878, there were tubercles on the ulnar side of the right arm and along the peroneal muscles of the right leg, with anæsthesia of those parts. He had also some tubercles upon the forehead. which, he explains, bled freely when torn. The skin of the affected parts is rough and thickened, especially over the face; upon the legs and arms there is desquamation; small, brown, elevated, and slightly anæsthetic patches of morphæ nigra are disseminated over the body, especially over the back. The mucous membranes of the nose and throat are affected; his voice is rough and hoarse; his nostrils obstructed by crusts. The conjunctivæ are injected. He describes the sensation of the flow of blood through the body as similar to the effect produced by the prick of needles or arrows. He suffers frequently from headache, heaviness, and drowsiness; can sleep at any time, day or night. Appetite and bowels normal.

He states that none of his relatives have been leprous. When 19 years of age he was engaged as a servant at the house of a leper—Eyvind Hansen Gulbrandsvik—in his native parish. Here he remained one year before coming to America, and he thinks he was then infected with the disease.

*Case IV.*—Hans Marcussen Dyrdal. Case observed and reported on by Professor Boeck, of Christiania (now deceased.) Steadily grew worse after that time, and died in 1878 of tubercular leprosy.—*Archives of Dermatology.*

